# Unusual Case of Testicular Tuberculosis

**DOI:** 10.5334/jbr-btr.1142

**Published:** 2017-03-29

**Authors:** Ikram El Hamrouni, Thierry Puttemans, Emmanuel Dardenne, Anne-Philippe Draguet

**Affiliations:** 1 Clinique Saint Pierre, Ottignies, BE; 2 Institute of Pathology and Genetics (IPG), BE

**Keywords:** miliary tuberculosis, BCG-therapy, epididymo-orchitis

## Abstract

We describe an unusual case of miliary tuberculous epididymo-orchitis following a BCG-therapy, mimicking malignancy at initial presentation. Genitourinary tuberculosis in a miliary pattern is rare and this case report emphasizes the importance of meticulous analysis of the patient’s clinical history combined with imaging findings in order to ensure an adequate diagnosis and treatment.

## Introduction

In the literature, only a few cases of tuberculous epididymo-orchitis, as complication of intravesical Bacillus Calmette-Guérin (BCG) therapy for urothelial cancer of the bladder, are reported. The radiological appearance of tuberculous epididymo-orchitis can be difficult given similarity to testicular tumour, testicular torsion, bacterial epididymo-orchitis or sarcoïdosis [1].

We describe a rare case with a characteristic appearance of tuberculous epididymo-orchitis in a miliary pattern which should alert the radiologist. This case report emphasizes the importance of meticulous analysis of the patient’s clinical history combined with imaging findings to ensure an adequate diagnosis and treatment.

## Case report

A 71-year-old Caucasian man presented to the emergency room complaining of left testicular swelling and tenderness for two weeks. The patient was afebrile and did not complain of any urinary symptoms. Past medical history was relevant: the patient had been diagnosed three years earlier with grade three papillary transitional cell carcinoma without involvement of the lamina propria and which had been treated by transurethral resection of the bladder tumour. After three years, he had a recurrence for which he had another resection and a treatment of intravesical instillation of Bacillus Calmette-Guérin. His last instillation was performed a month before his visit to the emergency department. Laboratory investigations revealed a slight elevated C-reactive protein (1.45 mg/dl – normal value 0.5 mg/dl) and A-FP (11 mcg/L – normal value < 8 mcg/l). The rest of blood and urine examinations were unremarkable.

**Figure 1:**
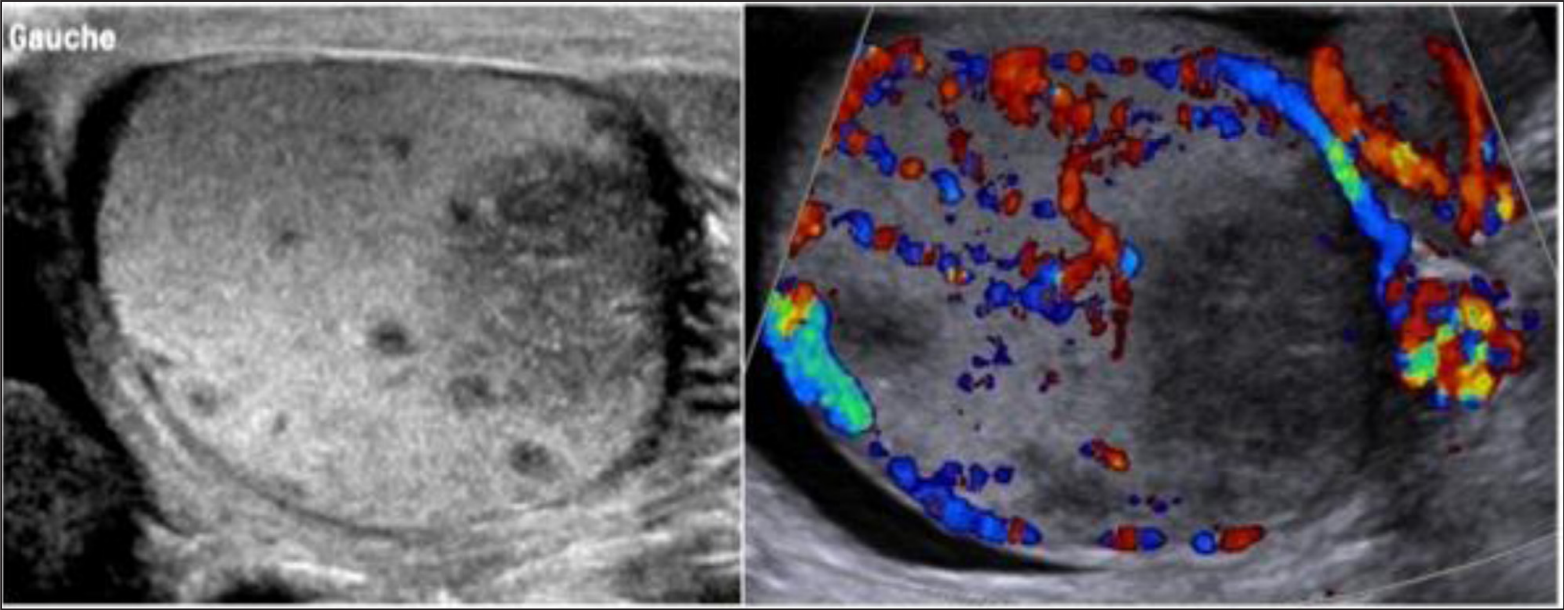
Grayscale and color Doppler of the left testis demonstrate hypoechoic avascular area of the hilum; the remaining testicular and epididymal parenchyma are more hyperechoic with flow detection.

**Figure 2:**
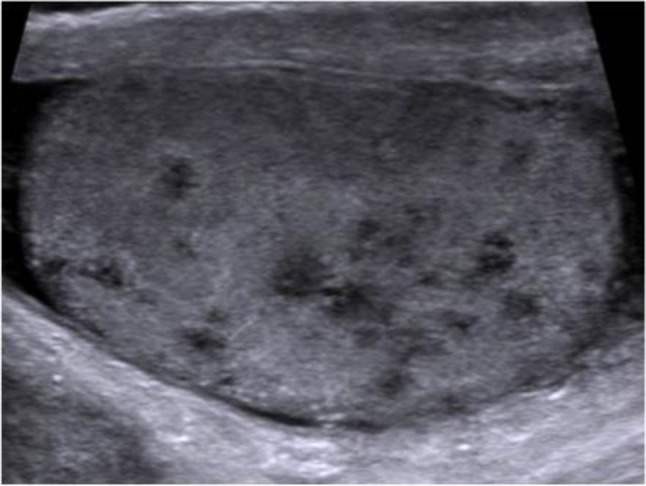
Longitudinal US image of the left testis shows multiple small hypoechoic nodules (< 2–3 mm) corresponding to TBC granulomas in a miliary pattern.

**Figure 3:**
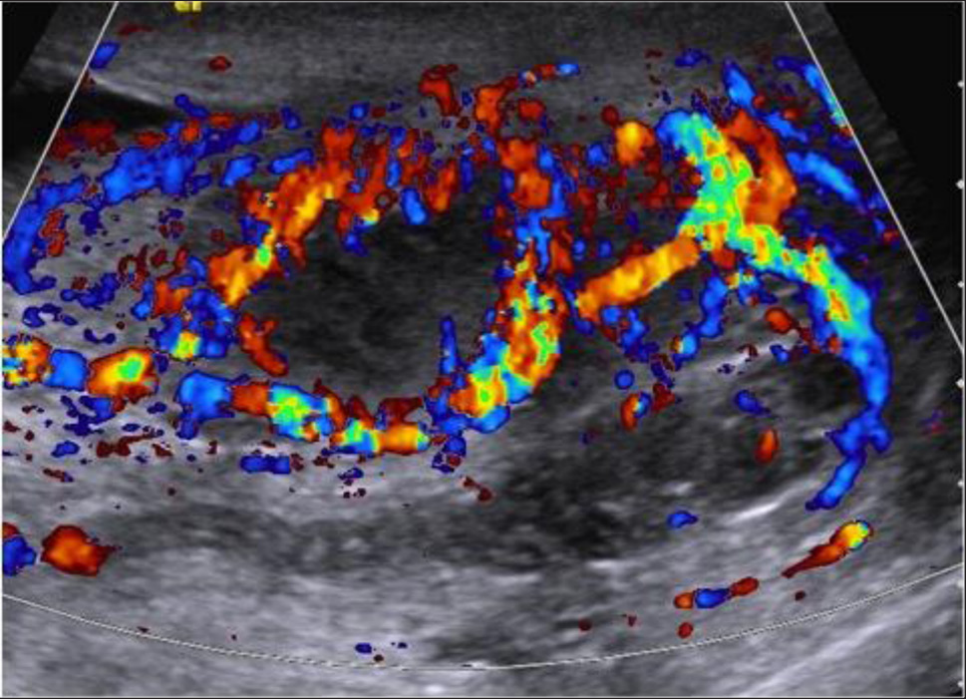
Longitudinal color Doppler image shows hypoechoic abscess of the hilum extending to the epididymis with increased flow signals in peripheral portion.

**Figure 4:**
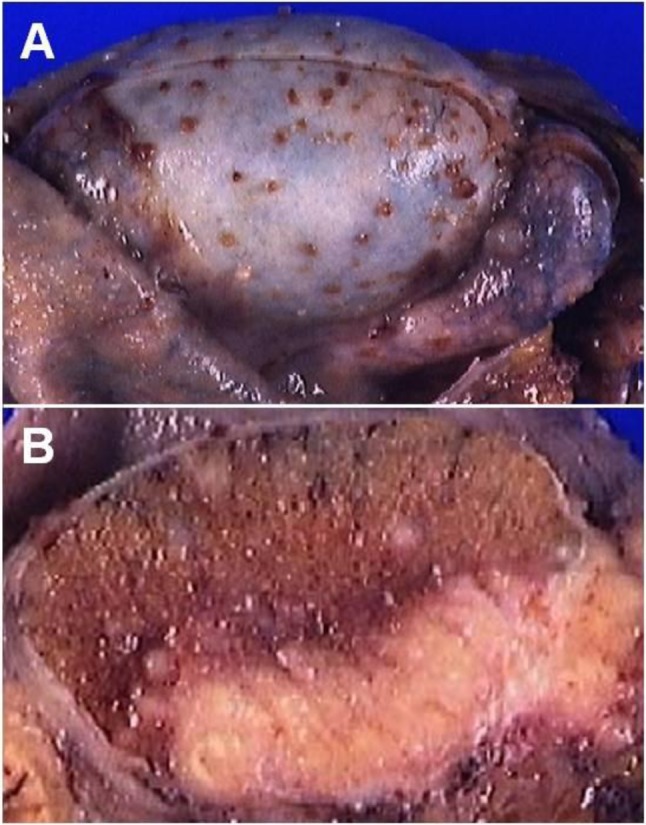
Histopathology specimens of the left testis. **(A)** Chirurgical specimen of left orchidectomy with many brownish nodules at the tunica albuginea and epididymis. **(B)** Cross section of left testicle showing caseating necrosis of the hilum and the epididymal head with muliple white millimetric nodules.

Color Doppler ultrasound of the scrotum performed the day of the visit by a junior resident showed a heterogenous hypoechoic and hypervascularized enlargement of the left epididymis, a round, hypoechoic, hypovascularized area involving the hilum (Figure 1) and multiple hypoechoic avascular micronodules (< 2–3 mm) scattered throughout the left testis (Figure 2). A small hydrocoele was present. A first diagnosis of testicular tumour was suggested and the patient was referred to the urologic department. Sonographic control, performed by the senior radiologist a week after the initial examination showed a global testicular hypervascularisation with large hilar round hypovascular area and multiple intratesticular hypoechoic micronodules. The epididymis was enlarged and hypervascularized with two hypoechoic hypovascular area. Based on these aspects, diagnosis of acute orchi-epididymitis with epididymal abscesses (Figure 3), early testicular necrosis of the hilum and multiple intratesticular granulomas suggestive of possible tuberculous origin was proposed and antibiotherapy was started. Successive US control performed during four months did not show any change. Due to the lack of response to antibiotherapy, an orchidectomy was performed. Anatomo-pathology revealed multiple necrotizing granulomas (Figure 4) with multinucleated giant cells. These lesions extend to the cord and epididymis and did not contain atypical cells. PCR was positive for Mycobacterium tuberculosis DNA. The culture was not contributive because of low initial quantity of mycobacteria. The patient had no other site of tuberculosis, and findings of chest CT and kidney US were normal.

## Discussion

In the usual tuberculous infection, scrotal infection with *Mycobacterium tuberculosis* is rare, occurring in about 7% of patients [1] although its incidence is increasing worldwide in association with human immunodeficiency virus infection. According to the Belgian Registry of tuberculosis, the incidence of genital TB in Belgium is nearly 1.7% of the reported cases in 2012. Scrotal infection usually starts at the tail of the epididymis, propagates to the entire epididymis and finally involves the testis [2].

In our case, the presence of miliary nodularity of the testis and the lack of response to conventional antibiotics should suggest the possible diagnosis of tuberculous epididymo-orchitis. Different echographic patterns have been described for tuberculous epididymitis and orchitis [1,3] including diffuse hypoechoic heterogeneous enlargement, diffuse hypoechoic homogeneous enlargement, and nodular hypoechoic heterogeneous enlargement. A miliary orchitis pattern is also possible, with multiple intratesticular hypoechoic nodules as observed in presented case. This miliary US pattern has been suggested to be characteristic of tuberculous orchitis [4]. Other typical findings include hydrocele, cutaneous swelling, extratesticular intrascrotal calcifications, abscesses, and scrotal sinus tract [1]. Only 11 cases of tuberculous epididymo-orchitis after bacillus Calmette-Guerin (BCG) therapy have been published according a review of literature by Parker et al. [5]. Granulomatous epididymo-orchitis is a rare complication of intravesical BCG therapy. Case reports show that epididymo-orchitis can occur up to 31 months after cessation of BCG therapy. After the diagnosis, we observed that the patient had presented with two episodes of micro-haematuria during his last instillations. This may mean the BCG invaded deeper into the bladder wall thus increasing the probability of an infectious side effect [5]. Clinically, the major differential diagnoses of tuberculous orchi-epididymis are testicular tumour, testicular torsion, bacterial epididymo-orchitis, and sarcoidosis [1]. Patients with tuberculous epididymo-orchitis following a BCG-therapy usually respond to anti-tuberculous therapy (300 mg of isoniazid and 600 mg of rifampicin daily for three to six months) [6]. The literature suggests that an orchidectomy is only required if there is abscess formation within the testis, otherwise anti-tuberculous therapy should be sufficient [7]. Often, the diagnosis is established after orchidectomy, performed because of a suspicion of testicular cancer. A biopsy should first be performed when epididymo-orchitis is part of the differential diagnosis [8].

## Conclusion

In a patient presenting with scrotal swelling, the US detection of miliary nodularity of the testis should alert the radiologist to the possible diagnosis of tuberculous epididymo-orchitis and consider Mycobacterium tuberculosis as the cause of epididymo-orchitis predominately after a BCG therapy and especially if there is no response to conventional antibiotic treatment.
